# DNA Fragmentation Simulation Method (FSM) and Fragment Size Matching Improve aCGH Performance of FFPE Tissues

**DOI:** 10.1371/journal.pone.0038881

**Published:** 2012-06-15

**Authors:** Justin M. Craig, Natalie Vena, Shakti Ramkissoon, Ahmed Idbaih, Shaun D. Fouse, Memet Ozek, Aydin Sav, D. Ashley Hill, Linda R. Margraf, Charles G. Eberhart, Mark W. Kieran, Andrew D. Norden, Patrick Y. Wen, Massimo Loda, Sandro Santagata, Keith L. Ligon, Azra H. Ligon

**Affiliations:** 1 Department of Medical Oncology, Dana-Farber Cancer Institute, Boston, Massachusetts, United States of America; 2 Center for Molecular Oncologic Pathology, Dana-Farber Cancer Institute, Boston, Massachusetts, United States of America; 3 Dana-Farber Cancer Institute, Boston, Massachusetts, United States of America; 4 Department of Pathology, Brigham and Women’s Hospital, Boston, Massachusetts, United States of America; 5 Hopital Pitie Salpetriere, Service de Neurologie Mazarin, Paris, France; 6 Department of Neurosurgery, Brain Tumor Research Center, University of California San Francisco, San Francisco, California, United States of America; 7 Division of Pediatric Neurosurgery, Acibadem University Medical Center, Istanbul, Turkey; 8 Department of Pathology, Acibadem University Medical Center, Istanbul, Turkey; 9 Department of Pathology, Children’s National Medical Center, Washington, District of Columbia, United States of America; 10 Department of Pathology, The University of Texas Southwestern Medical Center, Children’s Medical Center, Dallas, Texas, United States of America; 11 Departments of Pathology, Oncology and Ophthalmology, Johns Hopkins University School of Medicine, Baltimore, Maryland, United States of America; 12 Department of Pediatric Oncology, Dana-Farber Cancer Institute, Boston, Massachusetts, United States of America; 13 Department of Pediatrics, Boston Children’s Hospital, Boston, Massachusetts, United States of America; 14 Department of Neurology, Brigham and Women’s Hospital, Boston, Massachusetts, United States of America; 15 Center for Neuro-Oncology, Dana-Farber Cancer Institute, Boston, Massachusetts, United States of America; 16 Department of Pathology, Boston Children’s Hospital, Boston, Massachusetts, United States of America; 17 Harvard Medical School, Boston, Massachusetts, United States of America; Cleveland Clinic Lerner Research Institute, United States of America

## Abstract

Whole-genome copy number analysis platforms, such as array comparative genomic hybridization (aCGH) and single nucleotide polymorphism (SNP) arrays, are transformative research discovery tools. In cancer, the identification of genomic aberrations with these approaches has generated important diagnostic and prognostic markers, and critical therapeutic targets. While robust for basic research studies, reliable whole-genome copy number analysis has been unsuccessful in routine clinical practice due to a number of technical limitations. Most important, aCGH results have been suboptimal because of the poor integrity of DNA derived from formalin-fixed paraffin-embedded (FFPE) tissues. Using self-hybridizations of a single DNA sample we observed that aCGH performance is significantly improved by accurate DNA size determination and the matching of test and reference DNA samples so that both possess similar fragment sizes. Based on this observation, we developed a novel DNA fragmentation simulation method (FSM) that allows customized tailoring of the fragment sizes of test and reference samples, thereby lowering array failure rates. To validate our methods, we combined FSM with Universal Linkage System (ULS) labeling to study a cohort of 200 tumor samples using Agilent 1 M feature arrays. Results from FFPE samples were equivalent to results from fresh samples and those available through the glioblastoma Cancer Genome Atlas (TCGA). This study demonstrates that rigorous control of DNA fragment size improves aCGH performance. This methodological advance will permit the routine analysis of FFPE tumor samples for clinical trials and in daily clinical practice.

## Introduction

Tumor-specific genomic aberrations are of great diagnostic and prognostic value. In addition, these aberrations are increasingly useful in selecting targeted therapies for individual patients [1]. Current assays to establish copy number changes in clinical oncology are based on fluorescence in situ hybridization (FISH) and polymerase chain reaction (PCR) strategies designed to detect individual genomic alterations. However, large-scale cancer genome analyses continue to uncover specific aberrations in multiple cancers, and this, in turn, has driven the need for multiplex copy number testing in cancer research and clinical practice [2]–[4]. Genome-wide technologies to determine copy number changes such as array comparative genomic hybridization (aCGH) and single nucleotide polymorphism (SNP) arrays were among the first whole-genome technologies developed [5]. More recently, these technologies have been able to query the genome at intra-exon resolution and, as demonstrated in recent large-scale projects such as The Cancer Genome Atlas [4], can offer not only high-throughput analysis but also robust genome-wide copy number data.

Copy number analysis assays have been widely used in the research setting. Most of these basic research studies use frozen tumor samples that yield high-quality, intact DNA. The application of similar assays in clinical trials and in the routine clinical diagnosis of tumors has been unexpectedly slow, however. The greatest impediment to clinical implementation has been the technical challenges encountered during the processing and analysis of formalin-fixed paraffin-embedded (FFPE) samples, the mainstay of pathology department workflow. The inconsistent aCGH data that often results from FFPE samples is generally attributed to reduced DNA integrity. The relatively poor quality and variable results obtained from FFPE aCGH are particularly concerning because aCGH requires significantly more tissue than FISH or colorimetric *in situ* hybridization (CISH), both of which are performed routinely using FFPE specimens.

Early attempts at aCGH analysis of FFPE specimens were hindered because of inadequate sensitivity and specificity [6], [7]. Improvements in DNA extraction protocols [8]–[11], labeling techniques [12], and aCGH platforms [5], [13], [14] subsequently facilitated the analysis of FFPE samples in the research setting. To date, several studies have suggested that informative aCGH data can be generated from FFPE tissues [8], [9], [15]–[21], although our own prior experience and several reports in the literature indicate that one-third of FFPE specimens generate suboptimal aCGH results using standard methods [9]. This is particularly relevant for older specimens such as those used in retrospective analysis (e.g., clinical trials cohorts) [7], [15], [16], [19], [22].

Although the compromised integrity of DNA extracted from FFPE tissues has long been suspected as the source of the technical difficulties with FFPE aCGH, direct demonstration of this causal relationship and how to remedy it has proven challenging [7]. Several quality control (QC) metrics have been proposed for prospectively determining DNA suitability for aCGH. For each of these methods DNA degradation has generally been assessed using measurements of DNA size. Examples include: (1) multiplex-PCR to exclude DNA samples that fail to produce minimum size lengths; (2) gel electrophoresis to exclude DNA samples with average fragment size below a given minimum molecular weight; and (3) whole genome amplification (WGA) to exclude DNA samples that result in low DNA yields [9], [16], [21], [23], [24]. It is important to note that these studies assess DNA integrity prior to DNA labeling and subsequent hybridization. The specific conditions involved in DNA labeling - whether enzymatic- or chemical-based - cause additional fragmentation and physical modification of DNA [24], [25]. Therefore, any quality assessments performed prior to these steps do not evaluate the integrity of the DNA that is actually being hybridized to the array. Furthermore, these metrics help prevent assay failure without offering methods for improving the performance of samples known to contain suboptimal DNA.

If aCGH of FFPE specimens is to become feasible clinically, the process must be standardized to eliminate sample-to-sample variability as well as to significantly enhance both data quality and reproducibility [26], [27]. In this study, we examine the effect of DNA fragmentation on the outcome of aCGH analysis and describe a novel and powerful method designed to generate robust data and eliminate unpredictable quality variation among samples. Utilizing this method, we obtain significantly enhanced aCGH performance from both fresh and fixed sources of DNA. As proof of the versatility of our approach, we performed a rigorous demonstration on Agilent 1 M oligonucleotide arrays using a variety of FFPE glioma specimens of varying block age (1–15 yrs).

## Results

### Array Performance is Improved When Test and Reference DNA Samples Possess Similar Fragment Sizes

We first determined the effects of DNA fragment size on aCGH data quality. To do this, we conducted a series of self-hybridizations using a commercially available, high-quality genomic DNA (gDNA) sample that is a common reference standard in Agilent aCGH analyses (Promega, G1471, Madison, WI). The reference gDNA had a high molecular weight distribution at the outset (mode fragment length >10 kb). The sample was split into eight identical aliquots and heat-fragmented at 95°C for either 0, 5, or 10 minutes to generate a distribution of DNA sizes. The resulting DNA fragments demonstrated modes of 525, 225, and 140 bp. Each aliquot was labeled separately and paired in four combinations to create both size-matched and mismatched fragment pairs (matched pair: 225/225 and mismatched pairs: 525/225, 525/140, 225/140). These paired samples were then hybridized to Agilent 180 K feature arrays to model the variation in DNA fragment size commonly present in test and reference samples competitively hybridized to arrays.

**Figure 1:**
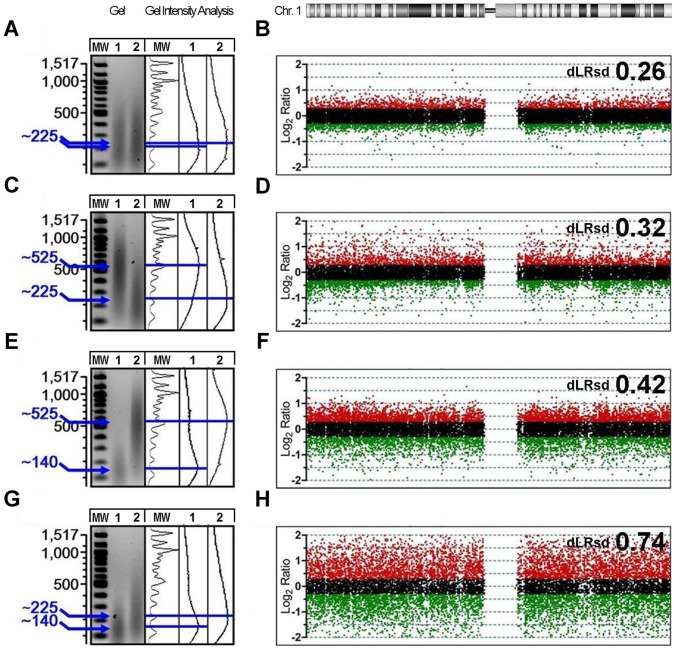
“Matching” DNA fragment size distributions are necessary for optimal aCGH data. (A,C,E,G) Agarose gel electrophoresis images and ImageJ gel intensity analysis plots of reference gDNA (Promega) after heat fragmentation. Mode fragment size is indicated in blue (bp) relative to DNA ladder. Heat times were adjusted to produce four mode fragment size combinations (225/225, 525/225, 525/140, 225/140). (B,D,F,H) Plot of results from chromosome 1 following self-hybridization of specific combinations of mode size. Differentially labeled aliquots (cy5/cy3) were coded as follows: green;log_2_ratio<-0.3, black;-0.3≤log_2_ratio≤0.3, red;log_2_ratio>0.3. Data quality was assessed by dLRsd on Agilent 180 K arrays.

Despite the initially intact and identical condition of the gDNA in each pair, three out of four self-hybridizations failed to achieve derivative log ratio spread (dLRsd) values less than 0.3, a primary QC metric and threshold for array data quality [19], [28] (Figure 1). The self-hybridization pair with matched DNA size distributions that had been exposed to identical fragmentation conditions resulted in a dLRsd of less than 0.3 (Figure 1A, B), indicating a hybridization likely to yield robust copy number data. The introduction of even moderate size mismatches (300 bp differential) was sufficient to introduce profound changes in final data quality, even when the mismatch resulted from an increase in fragment size (Figure 1C, D). Additional loss of data quality was noted when the difference in fragment sizes between the competitively hybridized DNA samples was further increased to 385 bp (Figure 1E, F). The magnitude of the size mismatch effect on data quality is not completely dependent on the magnitude of the size differential, however; as seen in the high dLRsd of the array data in Figure 1G, H, it is likely that decreased fragment size also adds complexity to the mechanism. These findings demonstrate that fragment size matching is critical for reducing the variability of array data quality even when using highly intact, optimal DNA samples.

### Determination of Optimal Mode Fragment Size in Size-matched Samples

**Figure 2:**
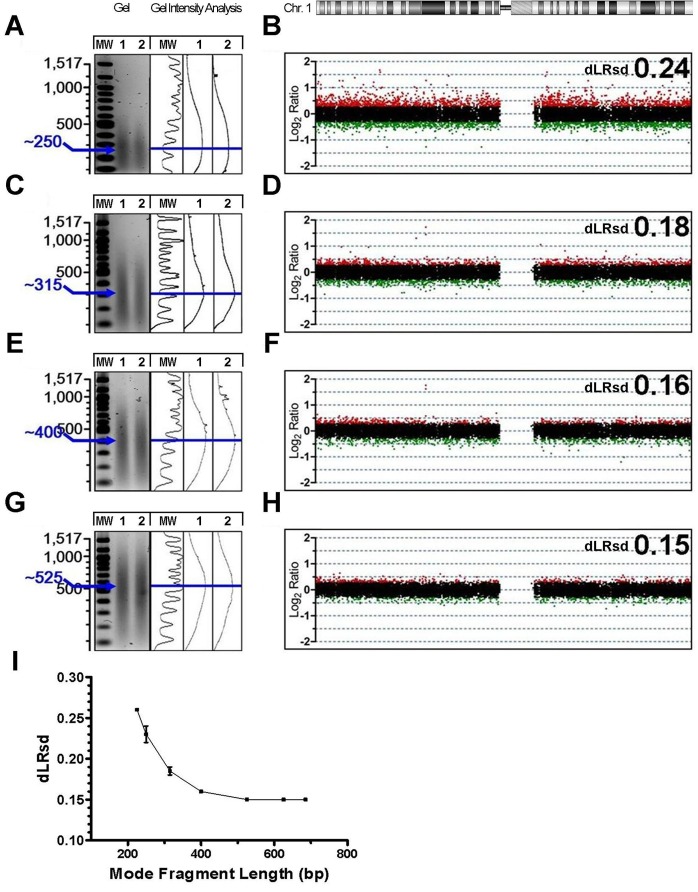
Determination of optimal size among matched DNA fragment size distributions. (A,C,E,G) Agarose gel electrophoresis of reference gDNA (Promega) aliquots after various heat fragmentation times shown adjacent to ImageJ gel analysis of same lanes, molecular weight indicated in bp. Mode fragment size of each smear, as measured with ImageJ, indicated in blue. (B,D,F,H) Agilent 180 K array results of self-hybridizations using reference gDNA (left) and characterized by matching fragment size distributions (B;250/250, D;315/315, F;400/400, H;525/525). Log_2_ ratios for signal intensities of differentially labeled aliquots (cy5/cy3) are plotted for probes corresponding to chromosome 1 (green;log_2_ratio<−0.3, black;-0.3≤log_2_ratio≤0.3, red;log_2_ratio>0.3). Data quality was assessed by dLRsd. (I) Mean dLRsd of duplicate (n = 5) or triplicate (n = 2) size-matched self-hybridizations representing seven fragment size distributions plotted by mode fragment length (225, 250, 315, 400, 525, 625, and 680 bp). Error bars indicate SEM.

Prior studies have indicated that experimental samples with fragment size distributions less than 300 bp may be a source of inconsistent aCGH performance [9], [16], [19], [24]. Given that matching fragment and reference DNA sizes improves results and might alter baseline performance, we sought to re-evaluate what the optimal fragment size might be under size-matched conditions. To test this we performed additional self-hybridizations using the reference gDNA sample and generated a spectrum of size distributions by varying heat fragmentation times. In total, 16 size-matched self-hybridizations representing seven unique size distributions (range ≈ 200–700 bp; mode fragment lengths ≈ 225, 250, 315, 400, 525, 625, and 680 bp), were measured in duplicate (n = 5) or triplicate (n = 2). In contrast to the size mismatched pairs shown in Figure 1C, D, E, F, G, H, all self-hybridizations between samples with matched fragment sizes yielded data within the acceptable range (dLRsd <0.3), regardless of length of the DNA fragments (Figure 2). We did however observe a significant correlation between decreased dLRsd and increased mode fragment size (r = −0.85, p = 0.015) (Figure 2) with optimal data quality achieved at mode fragment sizes greater than 400 bp (Figure 2I). Overall we observed that optimal aCGH data quality is produced with DNA fragment distributions of paired samples of similar sizes and mode fragment size greater than or equal to 400 bp.

### Tissue Sample DNA Responses to Heat Fragmentation Conditions are Intrinsically Variable and must be Determined Empirically

**Figure 3:**
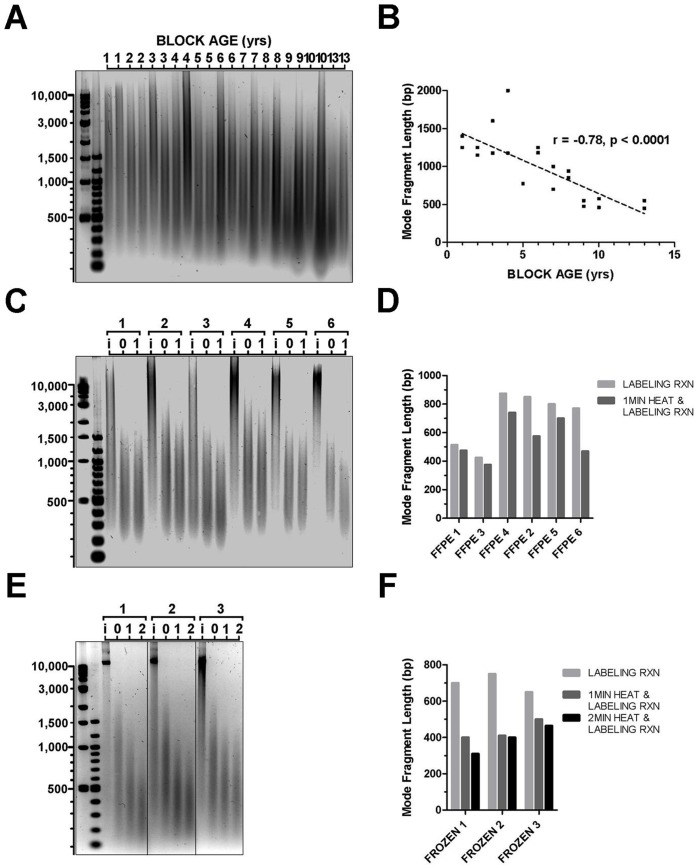
DNA fragmentation and thermodegradation are unpredictably variable. (A) Gel electrophoresis image of DNA extracted from 22 FFPE tissue specimens stored in paraffin from one to 13 years. (B) Mode fragment size of samples in (A) plotted by age of paraffin block, linear regression of data indicated by dashed line. (C) Gel electrophoresis image of DNA from six FFPE specimens intact prior to labeling (i), after ULS labeling only (0), or after ULS labeling plus 1 min heat fragmentation (1). (D) Mode fragment size of lanes marked 0 and 1 plotted for the six FFPE samples from the gel shown in (C). (E) Gel electrophoresis image of DNA from three frozen specimens with i, 0, and 1 indicating same conditions as in (C), and another samples after ULS labeling conditions plus 2 min heat fragmentation (2). (F) Plot of mode fragment size for lanes marked 0, 1, and 2 plotted for the three frozen samples in (E).

Utilizing our DNA extraction protocol with over 100 FFPE brain tumor specimens (block ages ranging from one to 15 years, all estimated to contain >50% tumor tissue) obtained from six different institutions, 100% of samples yielded DNA with average fragment sizes greater than 400 bp. Indeed, for most samples the fragment sizes were well above this size threshold and in agreement with general size ranges reported in other studies ([9], [19]). Agarose gel electrophoresis of 22 DNA extracts from FFPE tissue blocks ranging in age from one to 13 years confirmed this observation (Figure 3A). In fact, plotting the mode fragment size of each smear against block age reveals a statistically significant relationship (r = −0.77, p<0.0001) between advanced age and decreased fragment size (Figure 3B). Despite this relationship, our results support the conclusion that the initial (post-extraction) degradation of FFPE-derived DNA does not preclude obtaining fragment distributions within the optimal range (Figure 2), even among DNA samples isolated from archival specimens over ten years old.

Previous mechanistic studies of DNA thermodegradation describe significantly different rates of depurination and subsequent fragmentation in single versus double-stranded DNA [29], [30] and other studies have exposed the commonly overlooked role of nucleic acid degradation in standard PCR conditions [24], [25]. In light of these studies, we sought to identify whether the thermodegradation that occurs during labeling and other standard aCGH steps contributed to the variability in our aCGH results. Since the ULS Cy5 and Cy3 conjugates affect the electrophoretic mobility of DNA, we designed a simulated labeling reaction that exactly mimics the salt, solvent, and temperature conditions of the ULS labeling reaction. We then assessed DNA samples by gel electrophoresis following these simulated labeling conditions. Measured as the change in mode fragment size following heat fragmentation and/or labeling conditions, we observed significantly variable rates of thermodegradation across samples (Figure 3C, D, E, F), despite reproducibility in any given sample. Additionally, variable thermodegradation rates were observed even among samples of apparently similar initial size distribution, which confounded attempts to reliably predict the ultimate fragment size distribution of any given sample after heat fragmentation and labeling procedures based on the initial fragment size distribution of that sample. This intrinsic variability in DNA response to heat conditions in aCGH procedures was seen in all types of specimens, including fresh, frozen, and FFPE specimens alike (Figure 3C, D, E, F).

### Application of the Fragmentation Simulation Method (FSM) Allows Reliable Control of DNA Fragmentation Distributions and Improves Quality of aCGH Results

**Figure 4:**
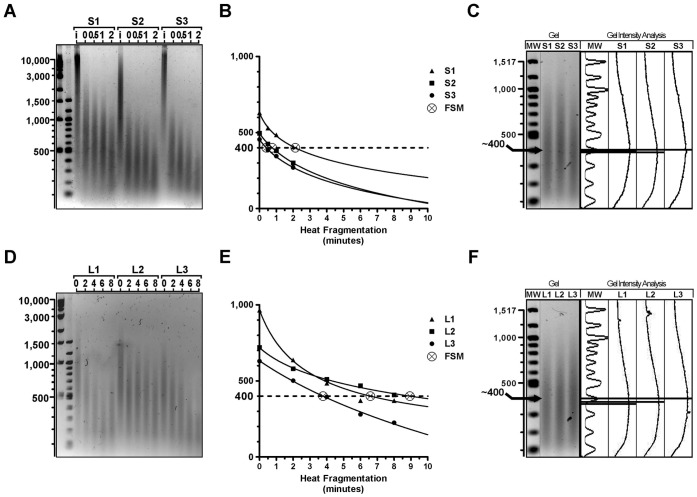
A Fragmentation Simulation Method (FSM) enables accurate prediction and precise control of labeled DNA fragment sizes. (A–F) Vertical axes indicate DNA bp. (A,D) Gel image of DNA from three FFPE specimens (A) or three frozen specimens (D) either intact, (i), after ULS labeling conditions only, (0), or ULS labeling conditions and 0.5, 1, 2, 4, 6, or eight minutes heat fragmentation (0.5, 1, 2, 4, 6, 8). (B,E) Utilizing mode fragment size of lanes in (A) or (D) as data points, FSM regression curves fit to data from each sample. Intersection with target size (dashed line) reveals FSM prediction for optimal time of heat fragmentation for each sample. (C,F) Agarose gel electrophoresis of samples in (A) or (D) after heat fragmentation for time predicted by FSM in (B) or (E) and ULS labeling conditions, shown adjacent to ImageJ gel analysis of same lanes. The mode fragment size of each smear, as measured with ImageJ, is indicated by arrows and solid horizontal lines.

The variability we observed in DNA thermodegradation rates suggested that the predefined fragmentation conditions used in published aCGH-FFPE protocols are unlikely to achieve the size uniformity required for optimal aCGH results. To increase the number of samples that yield high-quality aCGH data, we developed a Fragmentation Simulation Method (FSM) that allows fragmentation conditions to be tailored to individual samples using a single, standardized protocol. Observation of the time course of DNA thermodegradation in both fresh/frozen and FFPE DNA samples suggested that fragment size decay rates might best be modeled using an inverse power law as follows:
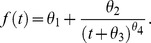
where *f(t)* is the mode DNA fragment size, in base pairs, of a sample’s fragment distribution immediately prior to hybridization (after a variable time of heat fragmentation and a simulated labeling reaction), *t* is time of heat fragmentation in minutes, while *θ*_1_, *θ*_2_, *θ*_3_, and *θ*_4_ are constant parameters unique for each sample. We experimentally determined data points (n≥4) by exposing aliquots of a DNA sample (≥50 ng each) to variable times of heat fragmentation (e.g. *t* = 0, 0.5, 1, and 2 minutes), followed by a simulated labeling reaction. The aliquots were then subjected to agarose gel electrophoresis and the open source ImageJ analysis software was used to determine the mode fragment size of each aliquot’s fragment distribution, *f(t)* (Figure 4A, D). An iterative least squares non-linear regression was then used to derive parameter values (*θ*_1_, *θ*_2_, *θ*_3_, and *θ*_4_) and fit a curve to the experimentally observed thermodegradation for each sample.

Once these parameters were determined, the completed model was used to predict the amount of heat fragmentation time, *t*, required to achieve an optimal mode fragment size, *f(t),* in each DNA sample (Figure 4B, E). Analysis of test samples subjected to heat fragmentation for a length of time indicated by the FSM and subjected to ULS labeling showed that the desired target fragment size distribution was attained (Figure 4C, F). Following the FSM and ULS labeling, samples are hybridized to arrays without further modification. Thus FSM provides a single, standardized protocol that accommodates the unique variation in the fragment size of an input DNA sample and its inherent thermodegradation rate.

### FSM Improves aCGH Quality and Reduces Sample-to-sample Variability in FFPE Samples

**Figure 5:**
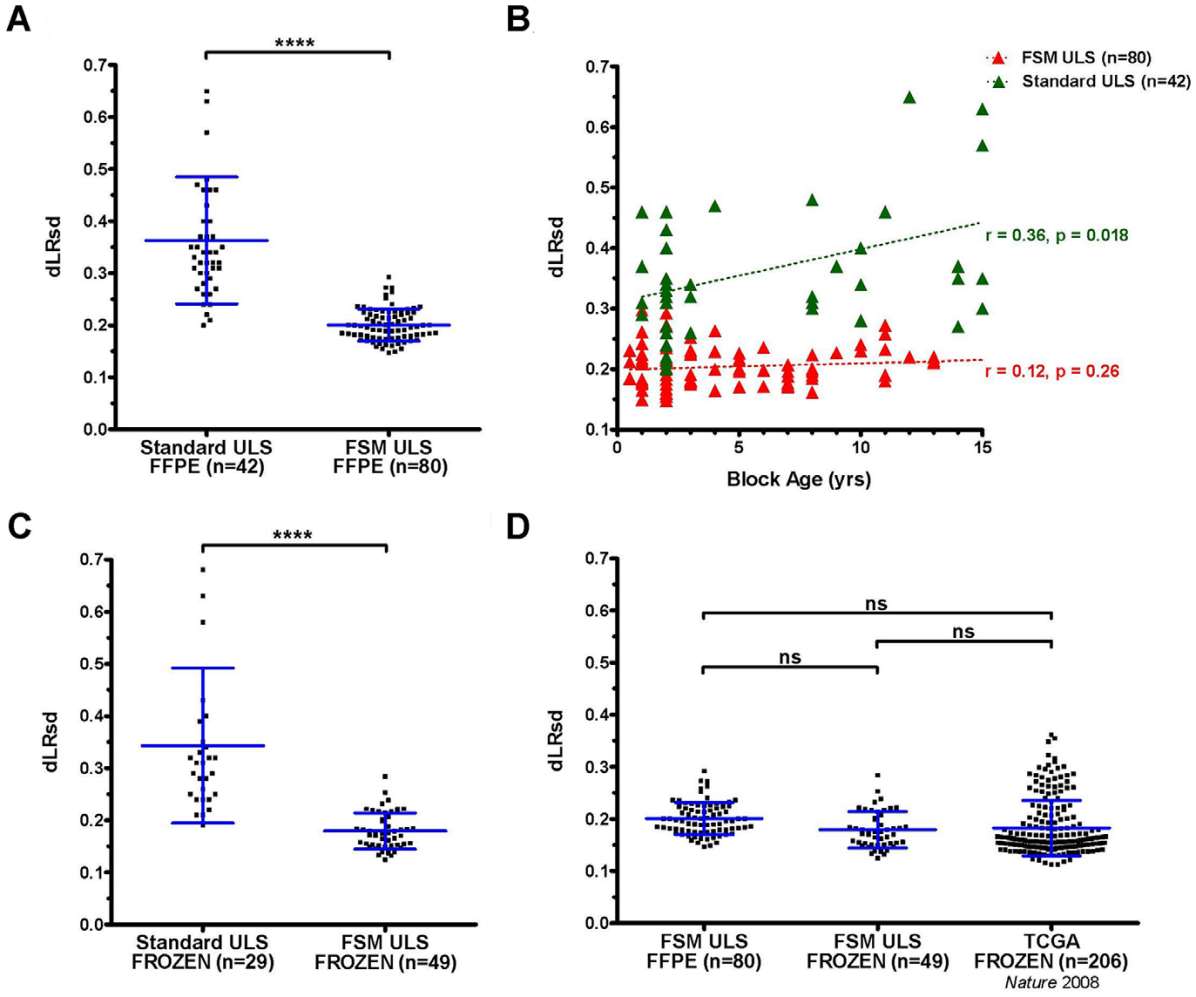
Application of FSM ULS method to FFPE samples creates equivalent results to those from fresh-frozen samples. (A) Plot showing dLRsd for 122 FFPE tumor specimens processed according to either standard ULS or FSM ULS protocols and analyzed on Agilent 1 M arrays. (B) Data quality (dLRsd) from (A) plotted by FFPE block age and method. Dashed lines indicate linear regression. Statistics indicate magnitude and significance of correlation between block age and aCGH data quality. (C) Quality (dLRsd) of Agilent 1 M aCGH data of 78 fresh-frozen tissue specimens or frozen tumorsphere cell cultures processed according to either standard ULS or FSM ULS protocols. (D) FFPE and Frozen FSM ULS subsets from (A) and (C) compared to 206 fresh-frozen GBM specimens analyzed on Agilent 244 k arrays from the glioblastoma TCGA study. Statistical significance was assessed by t test and ANOVA, (****;p<.0001, ns;p>0.05), and error bars indicate mean and standard deviation. Additional QC metrics data for all samples are provided in Table S2.

To determine whether the FSM method might improve the results obtained from both FFPE and non-FFPE tissue samples, we rigorously compared array data obtained using the FSM protocol with data obtained using the standard manufacturer’s ULS protocol. Hybridizations were performed using Agilent SurePrint stock arrays with a 1 million feature resolution. A diverse set of FFPE tumor specimens (n = 122), frozen tumor tissues (n = 7), primary tumorspheres and other tumor cell cultures (n = 71) were analyzed (Table S1). First, we assessed differences in the data quality generated by FFPE central nervous system (CNS) malignancies obtained from multiple institutions from blocks of various ages (one to 15 years). The quality of the array data processed according to the standard ULS protocol (n = 42, µ_dLRsd_ = 0.36, σ_dLRsd_ = 0.12) was inferior to that of samples processed according to the FSM ULS protocol (n = 80, µ_dLRsd_ = 0.20, σ_dLRsd_ = 0.03) with the difference reaching statistical significance (p<0.0001) as assessed by t and F tests (Figure 5A).

Noting significantly less variance in the quality of the FSM ULS subset, we chose to test whether the age of the tissue blocks and the resultant array quality are indeed related to one another, as previously suggested. In the standard ULS set, the correlation between increased sample age and lowered dLRsd was strong and significant (r = 0.36, p = 0.018), however this was not observed in the FSM ULS subset (r = 0.12, p = 0.26) (Figure 5B).

Since an optimal clinical laboratory protocol would ideally be the same for either fresh or fixed tissues and also because the ULS direct labeling approach has practical and experimental advantages over the commonly used enzymatic methods [24], we next examined the utility of the FSM ULS protocol using DNA isolated from either frozen tissue (n = 7) or frozen cells (n = 71) and Agilent 1 M feature arrays. As observed in the FFPE sample sets, the subset of frozen samples processed with the FSM ULS protocol (n = 49, µ_dLRsd_ = 0.18, σ_dLRsd_ = 0.04) demonstrated significantly (p<0.0001) higher quality and less variance than those processed according to the standard ULS protocol (n = 29, µ_dLRsd_ = 0.34, σ_dLRsd_ = 0.15) (Figure 5C). Finally, we compared quality across all of our FFPE and frozen sample sets as well as a previously published set of Agilent 244 k array data generated by The Cancer Genome Atlas project (TCGA) using fresh-frozen glioblastoma tissue specimens and traditional enzymatic DNA labeling (n = 206, µ_dLRsd_ = 0.18, σ_dLRsd_ = 0.05) [31]. One-way ANOVA and Tukey’s multiple comparison test reveal significant differences between the standard ULS subsets and each FSM subset as well as the TCGA subset (p<0.001). As depicted in Figure 5D, no significant difference was measured, however, between the FSM ULS FFPE subset, the FSM ULS frozen tissue subset, and the TCGA frozen tissue subset (p>0.05). Importantly, Figure 5 demonstrates that the FSM method enables the use of both fresh/frozen and fixed tissue sources for similarly robust, high-resolution aCGH data.

### DNA Fragment Size Matching Facilitated by the FSM Method is More Critical to Array Quality than Previously Identified Factors

**Figure 6:**
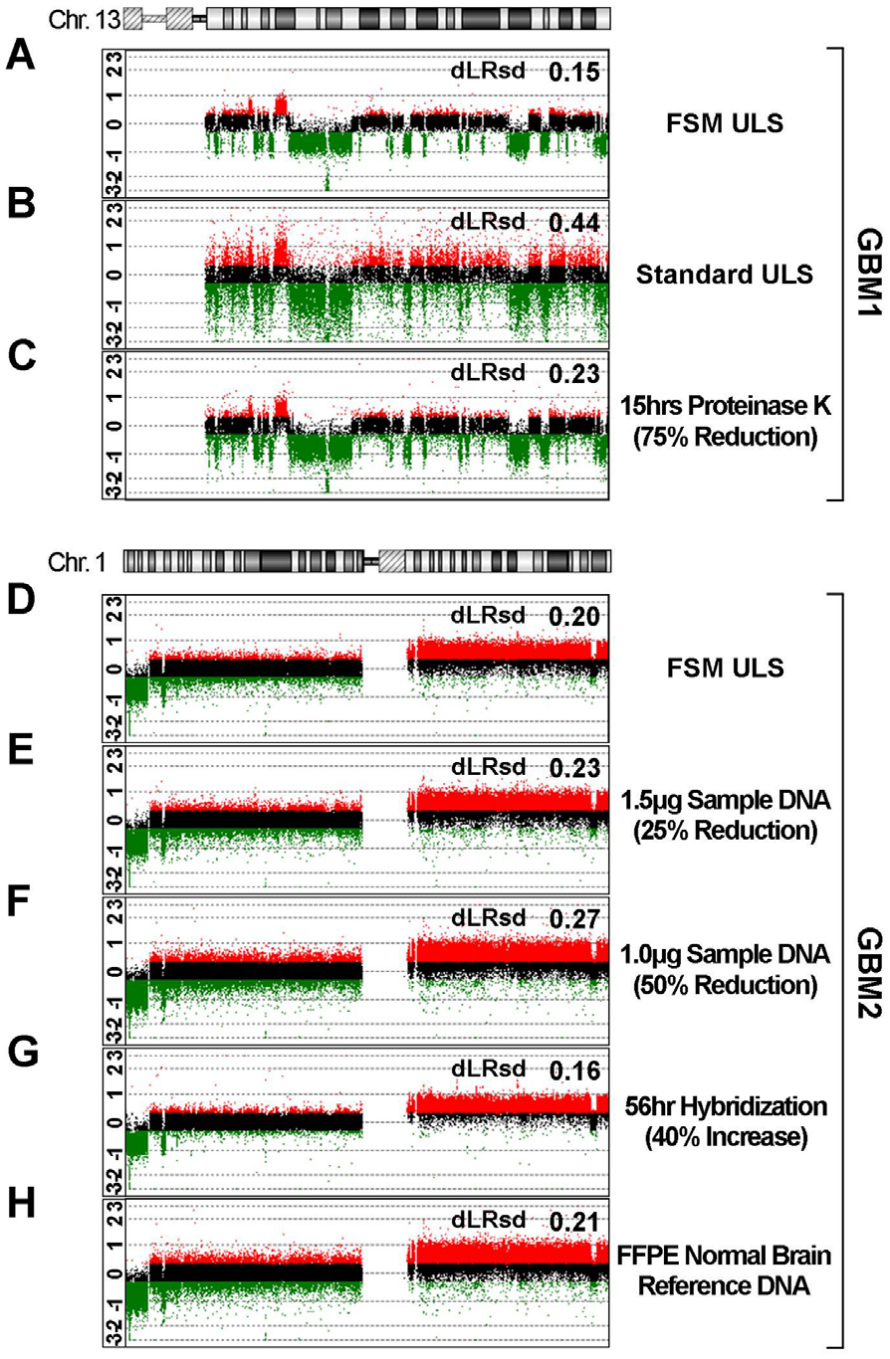
Size matching using FSM is a more critical determinant of array quality than other known variables. (A–H) Probe log_2_ ratio (signal intensity test DNA/signal intensity reference DNA) data plotted for a single chromosome (chr.13 or chr.1) from eight Agilent 1 M arrays (green;log_2_ratio<-0.3, black;-0.3≤log_2_ratio≤0.3, red;log_2_ratio>0.3). (A–C) Chromosome 13 plotted log_2_ ratios are representative profiles of three Agilent 1 M arrays of a single FFPE GBM specimen (GBM1) processed with the FSM ULS protocol (A), standard ULS protocol (B), or FSM ULS protocol after altered proteinase K digestion during DNA extraction (C) (plotted log_2_ ratio data for all chromosomes provided in Figure S2). (D–H) Chromosome 1 plotted log_2_ ratios are representative profiles of five Agilent 1 M arrays of a single FFPE GBM specimen (GBM2) processed using the FSM ULS protocol, with reduced DNA input in (E) and (F) (see Figure S3 and Figure S4 for detailed copy number analysis). Increased hybridization time (G) improved quality to a modest degree. Use of FFPE brain tissue as reference DNA (H) did not significantly improve results (dLRsd of 0.21 vs. 0.20 for standard reference).

Having demonstrated the highly significant contributions of FSM analysis and matched DNA fragment sizes to aCGH quality, we sought to further assess the relative effects of fragment size compared to other previously reported variables such as Proteinase K digestion time, array hybridization time, and concentration and source of DNA in array hybridization reactions. DNA from a single FFPE tumor specimen, GBM1 (characterized by complex and highly aberrant copy number changes involving single-copy gains, single-copy losses, and regions of homozygous deletion on chromosome 13), was processed under multiple conditions and assayed with Agilent 1 M feature arrays. Comparison of Figure 6A and 6B supports our previous assertions regarding the significant improvement of data quality enabled by the FSM. Compared with data obtained following the FSM ULS protocol (Figure 6A) the standard ULS protocol yielded a higher dLRsd value (0.44) (Figure 6B) that precluded accurate detection of copy number aberrations (Figure S1).

The duration of Proteinase K digestion during DNA extraction has frequently been identified as playing a critical role in the liberation of DNA from DNA-protein crosslinks and, consequently, it is thought to play a role in DNA labeling efficiency, hybridization, and resulting aCGH quality [8]–[12], [19]. The Agilent 1 M array data shown in Figure 6C was produced from GBM1 DNA exposed to only 15 hours of Proteinase K digestion rather than the 60 hour digestion in the typical FSM ULS protocol used for the data in (Figure 6A). The sample was otherwise processed according to an identical FSM ULS protocol. The effect of the reduced Proteinase K digestion was measurable by dLRsd (Δ_dLRsd_ = 0.08), although the data quality (dLRsd = 0.23) was well within recommended QC guidelines (dLRsd≤0.30) and aberrations across the whole genome were readily identified visually (Figure S2) and algorithmically. The chromosome 1 data shown in Figure 6D, E, F, G, H were generated using a single DNA sample from FFPE specimen, GBM2, and arrayed using five Agilent 1 M arrays. The data shown in Figure 6D represents baseline conditions (FSM ULS protocol, 2 µg each of GBM2 and Promega reference DNA, 40 hr hybridization). Single conditions were varied to generate the data shown in Figures 6E, F, G, H.

The tissue requirements of the assay are a critical factor and, as such, we sought to determine whether the FSM method would allow input of less DNA and still be able to generate robust results. The resultant data from Agilent 1 M array hybridizations with 25% and 50% reductions of DNA input (both tissue DNA and reference DNA) relative to the standard DNA input are shown in Figure 6E and 6F, respectively (data from additional hybridizations with 75% and 90% reductions of DNA input provided in Figure S3, DNA input ranging from 0.2–2.0 ug). While the expected negative trend is observed in the data quality of these arrays, it is important to note that even the dLRsd of the array hybridized with 1 ug DNA input (50% lower than standard) is still within an acceptable range (0.27). Perhaps more importantly, detection of copy number alterations by calling algorithms was 100% concordant with that of the baseline data shown in Figure 6D (concordance was measured as proportion of total aberrations detected with overlapping genomic position). This held true even on detailed copy number analysis of over 27 tumor specific aberrations (Figure S3). Examination of probe level sensitivity and specificity data for single copy gain/loss also showed highly reliable false positive/negative rates (FPR, FNR <0.20) at 1 ug of input DNA and reasonable performance even when only 0.2 ug of DNA was utilized (Figure S4).

Increased duration of hybridization is thought to positively impact the quality of array data and, because hybridization beyond 40 hrs may be of practical benefit in many clinical laboratory settings, we measured the effect of 40% more hybridization time (56 hrs). Indeed, the lower dLRsd (0.16) indicated improved quality as expected (Figure 6G), although detection algorithms did not yield additional information relative to the baseline data. We conclude that increasing hybridization times improved data quality and could actually be beneficial when tissue and DNA quantity are limited but that the magnitude of such improvement was less than that imparted by fragment size matching (see Figure 6E, F).

Finally, we sought to determine whether use of reference DNA of a more closely related tissue type and tissue fixation conditions might further improve results obtained from experimental samples. Data obtained from competitive hybridization of an FFPE brain tumor sample (GBM2 DNA from a glioblastoma) and genomic DNA isolated from FFPE “normal” brain tissue showed little suggestion of further improvement in data quality (dLRsd = 0.21).

**Figure 7:**
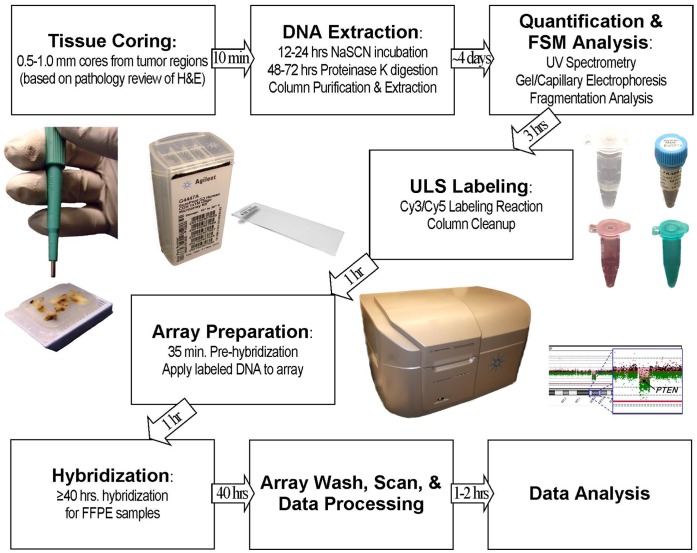
Overview of proposed methods for FSM ULS processing of FFPE specimens. Summary of methods and timeline for aCGH using the FSM method. Following DNA extraction, the workflow and protocol for preparation of fresh or frozen samples is identical to FFPE workflow shown.

**Figure 8:**
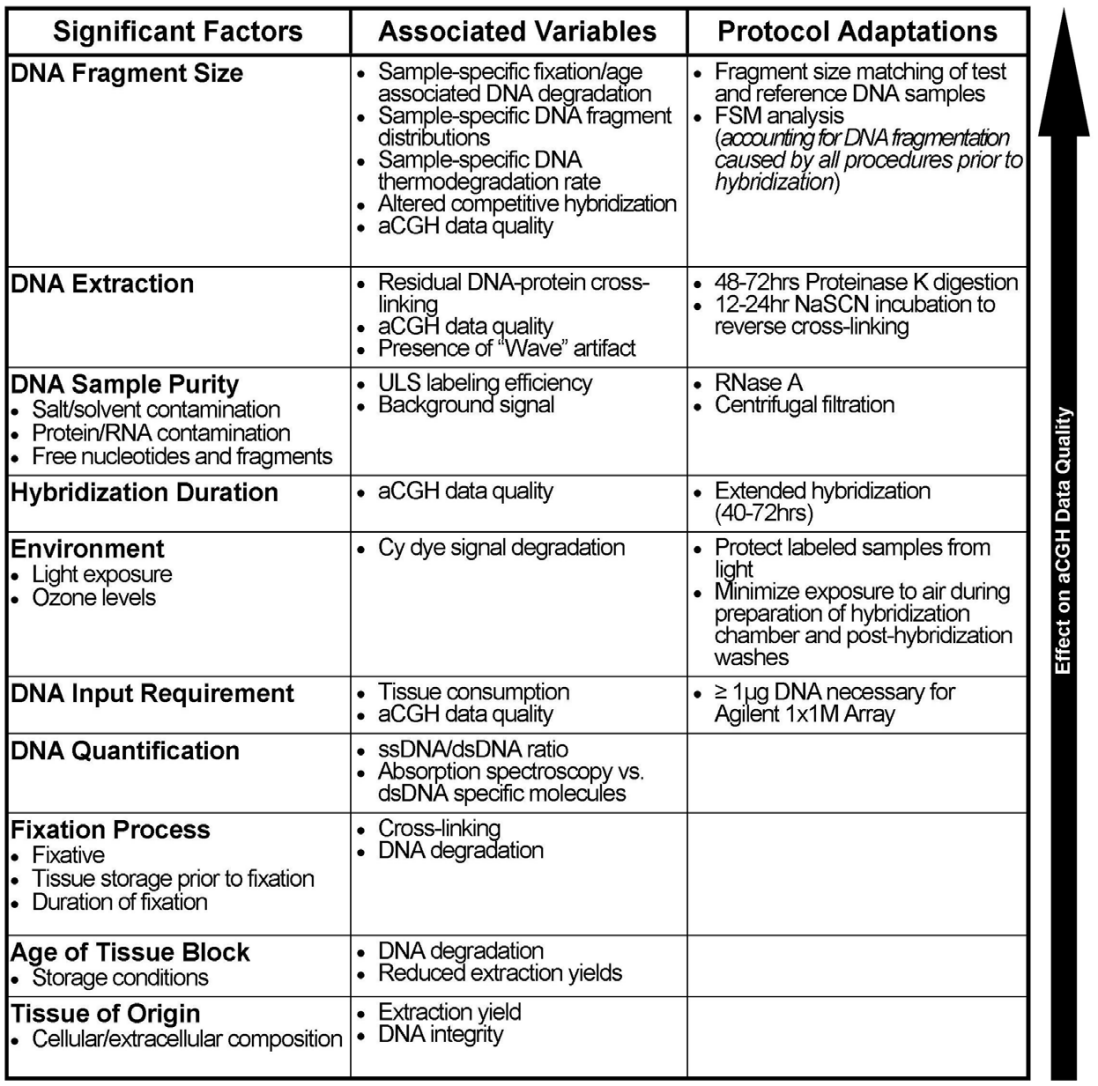
Predicted hierarchy of known variables contributing to aCGH data quality.

In summary, the use of FSM to match DNA fragment sizes (Figure 7) unveiled a hierarchy of factors that affect the performance of aCGH (Figure 8), and allow focused efforts to improve sample performance. Consequently, application of FSM expands the range of samples that can successfully be analyzed by aCGH.

## Discussion

Our results identify some of the major sources of aCGH variability and provide new methods for improving the data generated from suboptimal DNA specimens. By using a single source of high quality reference genomic DNA and carefully controlling DNA fragmentation, we were able to demonstrate that mismatched DNA fragment size distributions profoundly alter competitive hybridization under standard aCGH conditions more than previously suspected. These data are scientifically supported by previous biochemical studies which used short, fixed oligonucleotide probes to demonstrate that hybridization efficiency was inversely proportional to the length of the free (solution-side) end of the target strand in hybridizations. As a result, hybridization efficiency is significantly affected by DNA fragment length and the location of the hybridization along the length of the sequence [32]. When interpreted in the context of competitive hybridization, the findings of Peytavi et al. suggest that the competition of genomic DNA fragments may be significantly influenced by size-dependent hybridization efficiencies. As a fundamental assumption underlying all CGH technology, equivalent hybridization properties of differentially labeled DNA fragments are necessary if concentration (i.e. copy number) is to be accurately reflected by signal intensity at equilibrium [33]. Therefore, we hypothesize that - by matching the DNA fragment sizes of both samples - we have presumably minimized differences in hybridization efficiency and thereby promoted improved data quality.

Additionally, we speculate that matching DNA fragment size within an optimal size range further increases the proportion of fragments that are viable hybridization targets and therefore increases the effective target concentration, driving the hybridization towards thermodynamic equilibrium. This effect can explain the high quality results generated by the FSM ULS method and why it also allows use of less sample DNA (Figure 6F), similar to the manner in which extended hybridization improves data quality (Figure 6G) by allowing the reaction to proceed closer to equilibrium.

Regardless of mechanism, the empirically demonstrated effect of matching fragment sizes in competitively hybridized DNA samples enabled us to apply the FSM ULS protocol and achieve the robust aCGH data reported here. The utility of FSM ULS also supports the substantial predictive power of prospective quality control assays [9], [16], [23], [24]. Since these latter assays based their sample selection criteria on indirect measures of DNA fragment size, each enabled a beneficial selection of samples with more appropriate and homogenous DNA fragment size distributions. We suspect that the percentage of samples that failed to yield meaningful aCGH data in each study can be explained by unaccounted DNA fragmentation occurring during labeling, as well as by variable thermodegradation rates intrinsic to the sample (Figure 3), and/or dissimilar reference DNA fragment distributions. DNA fragment size matching is also likely to have contributed to improved aCGH quality obtained in a recent study advocating application of DNase I fragmentation and enzymatic labeling [19]. Notably, this study is among several recent reports that have also attributed their improved aCGH performance with FFPE tissues to the labeling of increased amounts of sample DNA (as much as 5 µg for an Agilent 244 k array), a practice that has been cited as necessary to overcome the negative effects of the compromised template DNA [34], [35]. While increasing the amount of DNA in the reaction may achieve similar results, the use of such large amounts of DNA is not generally practical for application to standard clinical samples where the amount of tissue available is limited, and current trends and future technologies will likely necessitate use of only nanogram amounts of DNA. While our method as described should allow the widest adoption by labs, we anticipate that reductions in DNA requirements may be achieved with the FSM and other methods through use of low-sample volume capillary gel electrophoresis systems in the size modeling step. Additional reductions may come from the use of lower resolution arrays that are generally still of sufficient resolution to identify the majority of clinically relevant cancer aberrations.

Another likely source of improved results in the methods described is our preferred use of the chemically based ULS labeling method over enzymatic methods. Conceptually, ULS labeling is less affected by fixation-associated artifacts such as DNA cross-linking and DNA fragmentation. The ULS technology, which employs a platinum-based chemical reaction, adds Cy3 and Cy5 conjugates directly to the sample DNA at the N^7^ position of guanine bases, and also is independent of DNA strand length [12], [36]. In contrast, enzymatic labeling further degrades the DNA during required denaturation steps [25], reduces the complexity of the original genomic template, and therefore may introduce bias in downstream copy number data [12]. Yet despite the advantages of ULS labeling, use of this labeling approach is not as widely reported, particularly with intact DNA sources such as fresh tissues or blood [19]. We observed marked variation in performance of standard ULS labeled samples, consistent with the outcomes reported by Hostetter et al. As a result, we believe that application of the FSM method was integral to the successful hybridization of relatively intact DNA because the appropriate fragmentation time required by a given sample was more variable than that of the FFPE derived samples (Figure 4E). To our knowledge, our study is one of the first large-scale studies to report the successful application of ULS labeling to high-resolution aCGH analysis of non-FFPE as well as FFPE DNA sources. Our methodology may therefore allow a wider use of ULS technology, which offers distinct benefits of speed and simplified sample preparation across cancer and non-cancer applications (Figure 7).

With regard to the fundamental suitability of FFPE samples for whole-genome analyses, our results with the FSM ULS protocol suggest that FFPE DNA is not damaged in any way that irreversibly affects aCGH performance, but methods to account for the decreased DNA fragment size encountered must be more routinely implemented. The correlation between FFPE block age and increased fragmentation is consistent with the lower success rates previously reported with older samples when fragment size was not carefully controlled, but our results suggest that recommendations that samples older than 10 years of age should be excluded from research or clinical analysis need to be reevaluated. Future analysis of samples beyond 15 years of age may aid in determining whether an upper age limit might exist for FFPE specimens analyzed by aCGH using FSM or other methods. Notably, while the Agilent stock 1 M feature array offers extremely high resolution and a genome wide median probe spacing of 2.1 kb, the enhanced resolution confers greater sensitivity to both true copy number alterations as well as “noise” when compared with lower resolution arrays such as the Agilent 244 k array [34], [37]. The choice of the Agilent 1 M array for quality comparisons therefore represents a significantly stringent standard for any aCGH method and the fact that we achieved uniform dLRsd below 0.3 over large and diverse samples sets from multiple international institutions using a wide range of fixation conditions argues again that the array type and other variables are potentially minor variables in array performance relative to sample preparation and hybridization conditions.

In developing the FSM methodology we optimized the protocol to use commonplace and affordable laboratory equipment and to not require complex procedures. Although this method uses heat fragmentation, it is likely that other methods that allow greater control over matched DNA fragment distributions could also be used successfully. Sample methods that utilize Covaris, restriction enzyme, or size selection approaches would be useful to compare to the results from heat fragmentation reported here. Application of FSM methodology to standardization of genomic DNA ensures that powerful FFPE-compatible diagnostic laboratory tools can more easily be implemented into routine clinical use. Perhaps more exciting is the possibility that the FSM approach of modeling nucleic acid fragmentation to predict downstream fragment sizes also may have utility for other hybridization-based reactions, such as Affymetrix SNP arrays or hybrid capture methods commonly used in next generation sequencing. While the studies here should allow clinical applications of aCGH to proceed, future studies will likely focus on further reducing the complexity, amount of input DNA, and time required to conduct aCGH analysis.

## Materials and Methods

### Tissue and Cell Line Specimens

Formalin-fixed paraffin embedded tissue specimens (n = 122) and fresh-frozen tissue specimens (n = 7) were obtained from six separate institutions under de-identified excess tissue protocols approved by institutional review boards at each institution (Boston Children’s Hospital, Boston, MA (CHB); Brigham and Women’s Hospital, Boston, MA (BWH); Children’s Medical Center of Dallas, Dallas, TX (CMCD); Johns Hopkins Medical Institute, Baltimore, MD (JHMI); Children’s National Medical Center, Washington, D.C. (CNMC); and Marmara University Medical Center, Istanbul, Turkey (IST)). The IRB/ethics committee of each institution specifically waived the requirement for consent for these studies. All FFPE tissue specimens were human CNS malignancies or “normal” brain controls from non-neoplastic epilepsy specimens. Tumor samples were estimated to contain >50% tumor nuclei in all cases. Diagnoses were established by histologic examination according to the criteria of the World Health Organization classification by two neuropathologists (K.L.L. and S.S.). Primary glioma and other brain tumor cell lines were obtained either from the Dana-Farber Cancer Institute/Brigham and Women’s Hospital Living Tissue Bank (DF/HCC) (n = 64) or from the University of California San Francisco (UCSF) (n = 7).

### Reference DNA

Commercial reference genomic DNA (created from fresh peripheral bloods pooled from five to seven healthy, karyotypically normal individuals) was purchased from Promega (cat. no. G1471/G1521, Madison, WI).

### DNA Extraction

#### FFPE tissues

Genomic DNA was extracted from FFPE tissues using a protocol similar to that previously described [9]. Briefly, 1 mm cores (two to five cores total) or 20 µm sections (three to five sections total) were taken from regions estimated to contain greater than 50% tumor cells based on previous pilot studies showing accurate detection of single copy gains and losses in samples with >40% tumor nuclei by pathologist estimate of H&E slides. Cores or sections were placed in sterile nuclease-free microcentrifuge tubes and paraffin was removed by treating the tissue in (1.2 ml) xylene. Samples were rinsed twice with 1.2 ml 100% ethanol and allowed to dry at room temperature before the addition of 0.9 ml 1 M NaSCN and overnight incubation at 37°C. After 12–24 hrs, samples were rinsed twice in 0.9 ml 1X PBS. 0.34 ml of Buffer ATL (Qiagen, QIAamp DNA FFPE Tissue Kit cat. no. 56404, Valencia, CA) and 40 µl of Proteinase K (20 mg/mL) (Qiagen, cat. no. 19131) were added and samples were incubated in a thermomixer (Eppendorf, cat. no. 022670000, Hamburg, Germany) set at 56–58°C and 450 rpm. An additional 40 µl Proteinase K was added every 8–12 hrs for a period of 48–72 hrs. Samples were allowed to cool to room temperature before the addition of 10–20 µl RNase A (100 mg/mL) (Qiagen, cat. no. 19101) and a 5–10 minute incubation at room temperature. After adding 400 µl of Buffer AL (Qiagen QIAamp DNA FFPE Tissue Kit), samples were placed in thermomixer at 60°C for 10 minutes. 440 µl of 100% ethanol was added and each sample was split between two QIAamp MinElute Columns (Qiagen QIAamp DNA FFPE Tissue Kit). Following successive washes with 500 µl Buffer AW1 (Qiagen QIAamp DNA FFPE Tissue Kit) and 500 µl 80% ethanol, DNA was eluted in 50–100 µl H_2_O.

#### Frozen tissues and cells

Genomic DNA was extracted from frozen tissue and cell line samples using the DNeasy Blood & Tissue Kit (Qiagen, cat. no. 69504). The manufacturer’s protocol was utilized with the inclusion of the optional RNase A treatment and the replacement of Buffer AW2 with 80% ethanol. DNA was eluted in 100–200 µl H_2_O.

### FSM Analysis

Prior to FSM analysis, all DNA samples were concentrated using 30 K MWCO Amicon Ultra Centrifugal Filter Units (Millipore, cat. no. UFC503096, Billerica, MA). The use of these filters also removes ssDNA and dsDNA fragments of 50–60 nt in length, and facilitates the serial dilution of residual salt and/or solvent in the purified DNA samples. Concentrated DNA samples were quantified by absorbance spectroscopy with a NanoDrop 1000 (Thermo Fisher) and diluted to working concentrations specific to Agilent aCGH array-dependent requirements (e.g. 125 ng/µL for 1 M arrays or 62.5 ng/µL for 180 K arrays). Briefly, a minimum of 240 ng DNA was removed from each sample and brought to a total volume of 32 µl with H_2_O. This solution was then split into 8 µl aliquots in the same 200 µl PCR tubes that were to be used for the ULS labeling reactions. These four aliquots were heat-fragmented at 95°C in a PCR thermocycler for either 0, 0.5, 1, or 2 minutes (FFPE samples) or 0, 2, 4, or 6 minutes (frozen tissue/cells) immediately followed by a 4°C cycle of at least 4 minutes duration. Using volume and composition proportions consistent with the 1 M array ULS labeling reaction, 2 µl of ULS labeling simulation solution (50% 10X Labeling Solution (Agilent Technologies, Genomic DNA ULS Labeling Kit cat. no. 5190-0419, Santa Clara, CA), 25% 20 mM NaCl, 25% DMF) was then added to each of the four aliquots before simulated ULS labeling reaction conditions were initiated (30 min at 85°C then ≥10 min at 4°C in PCR thermocycler). Sample aliquots were combined with 4 µl Orange (6×) Gel Loading Dye (New England Biolabs, cat. no. B7022S, Ipswich, MA) and loaded on 1.5% agarose 1X TBE gels prior to electrophoresis at 100–120 V. Gels were stained with GelRed Nucleic Acid Stain (Phenix Research Products, cat. no. RGB-4103, Candler, NC). Utilizing open-source ImageJ analysis software (U. S. National Institutes of Health, Bethesda, MD), the mode fragment size of each aliquot was approximated by referencing the maximum intensity of each smear with the bands of a 100 bp DNA Ladder (New England Biolabs, cat. no. N3231S). The fragmentation of each sample was modeled using this data in combination with Equation 1 and JMP 8 analysis software (SAS Institute Inc., Cary, NC), and an optimal heat fragmentation time was determined.

### Array CGH

#### FSM ULS

Purified DNA extracts from FFPE tissues, frozen tissues, and frozen cells were heat fragmented as indicated by FSM analysis. Subsequently, ULS labeling (Agilent Technologies, Genomic DNA ULS Labeling Kit cat. no. 5190-0419, Santa Clara, CA) was performed according to the manufacturer’s suggested protocol. Briefly, 2 µg DNA from each sample was combined with 2 µL ULS-Cy5 Reagent (Genomic DNA ULS Labeling Kit) and 2 µL 10X Labeling Solution (Genomic DNA ULS Labeling Kit) prior to 30 min at 85°C and ≥10 min at 4°C in a PCR thermocycler. An equal mass of either male or female reference DNA was heat-fragmented according to FSM predictions and then labeled with the ULS-Cy3 Reagent (Genomic DNA ULS Labeling Kit). Unincorporated dye was removed using Genomic DNA Purification Modules (Agilent Technologies, cat. no. 5190-0418). The entire volumes of the Cy5-labeled sample DNA and the Cy3-labeled reference DNA were combined together with 37.8 µL H_2_O, 50 µL Cot-1 DNA (Invitrogen, cat. no. 15279-011, Carlsbad, CA), 5.2 µL 100X Blocking Agent (Agilent Technologies, Oligo aCGH Hybridization Kit cat. no. 5188-5220), and 260 µL 2X Hi-RPM Hybridization Buffer (Agilent Technologies, cat. no. 5190-0403) before denaturation (3 min at 95°C) and pre-hybridization (30 min at 37°C). 130 µL Agilent-CGHblock (Agilent Technologies, cat. no. 5190-0421) was added to each hybridization solution before 490 µL of the combined solution was applied to a gasket slide (Agilent Technologies, cat. no. G2534-60003). A 1×1 M SurePrint G3 Human CGH Microarray (Agilent Technologies, cat. no. G4447A) was paired with each gasket slide in a SureHyb Enabled Hybridization Chamber (Agilent Technologies, cat. no. G2534A) and the differentially labeled DNA samples were hybridized (65°C) to the microarray for 40–72 hrs in a hybridization oven (Agilent Technologies, cat. no. G2545A). During hybridization the slides were rotated at 19 rpm.

#### Standard ULS

DNA extracted from FFPE tissues was not subjected to additional fragmentation prior to ULS labeling. The intact DNA extracted from frozen tissues and cells, as well as reference DNA samples, were heat fragmented for ten minutes as suggested by the manufacturer’s standard ULS protocol. The remainder of both labeling and hybridization procedures was identical to those of the FSM ULS method.

#### Self-Hybridizations

Single sample self-hybridizations utilized male reference genomic DNA (Promega, G1471, Madison, WI). DNA was suspended in nuclease-free H_2_O using 30 K MWCO Amicon Ultra Centrifugal Filter Units. 500 ng aliquots were heat-fragmented (95°C) for varying lengths of time and then differentially labeled with ULS-Cy3 Reagent and ULS-Cy5 Reagent before hybridization to 4×180 K SurePrint G3 Human CGH Microarrays (Agilent Technologies, cat. no. G4449A), and according to the manufacturer’s standard ULS protocol.

#### Microarray washing, scanning, and feature extraction

Microarrays and gaskets were disassembled at room temperature in Wash Buffer 1 (Agilent Technologies, cat. no. 5188-5221) and quickly moved to a second dish containing Wash Buffer 1 and a stir bar rotating at speed sufficient for gentle agitation of the liquid’s surface. After 5–30 minutes, slides were moved to a dish containing Wash Buffer 2 (Agilent Technologies, cat. no. 5188-5222) and a stir bar and agitated at 37°C for 1 minute. Slides were then washed in anhydrous acetonitrile (Sigma-Aldrich, cat. no. 271004, St. Louis, MO) for 10–15 sec before being removed and placed in a slide holder (Agilent Technologies, cat. no. G2505-60525) with an Ozone-Barrier Slide Cover (Agilent Technologies, cat. no. G2505-60550). Microarrays were scanned immediately with a DNA Microarray Scanner (Agilent Technologies, cat. no. G2505C) at 3 µm resolution. Scanned images were processed using Agilent Feature Extraction v10.7 and FE Protocol CGH_107_Sep09. Quality control dLRsd statistics were recorded as reported in the QC Metrics file generated by the software.

### Data Analysis

Copy number analysis was performed using the DNA Analytics module of Agilent Genomic Workbench 6.5. Log_2_ ratios were corrected for a periodic “wave” artifact that correlates with GC content using the software’s GC correction tool with a GC window size of 2 kb. The ADM-2 algorithm was used with a threshold of 6.0 to detect significantly aberrant genomic regions and detected regions were filtered for those spanning more than five probes (∼10 kb) with an average absolute log_2_ ratio >0.3. Array data has been published in compliance with MIAME 2.0 guidelines and deposited in the publicly available ArrayExpress database.

## Supporting Information

Figure S1 **FSM ULS probe level data demonstrates greater sensitivity and specificity than Standard ULS probe level data.** Female FFPE tumor DNA from sample GBM1 hybridized with normal male reference DNA (Promega) on Agilent 1 M arrays using either the FSM ULS or Standard ULS protocols. Log_2_ ratio data from X chromosome (XX/XY) and chromosome 8 (copy neutral) are compared for each array. A) Receiver operating characteristic (ROC) curves plot sensitivity and specificity across a range of log_2_ ratio thresholds and indicate aberrant (X chromosome) probe values are more readily distinguished from non-aberrant (chromosome 8) probe values in FSM ULS data than in Standard ULS data (AUC indicates area under respective ROC curve). B,C) Given optimized log_2_ ratio thresholds defined by ROC analysis (blue), log_2_ ratio frequency distributions are plotted and false positive rate (FPR) and false negative rate (FNR) are calculated. FPR is defined as proportion of copy neutral (chr8) probe values incorrectly classified as aberrant and FNR is defined as proportion of aberrant (Xchr) probe values incorrectly classified as copy neutral.
(TIF)

Figure S2 **Whole genome view of Agilent 1** **M array data for FFPE sample GBM1 prepared by FSM versus standard ULS methods.** Log_2_ ratios plotted for three Agilent 1 M arrays hybridized using either the FSM ULS protocol (left), the standard ULS protocol (middle), or the FSM ULS protocol and DNA extracted with reduced duration Proteinase K digestion (right) as in Figure 6A–C (green; log_2_ratio<−0.3, black;−0.3≤log_2_ratio≤0.3, red;log_2_ratio>0.3). FSM methods yield lower noise across the whole genome compared to standard ULS even with shorter Proteinase K digestion.
(TIF)

Figure S3 **FSM ULS protocol enables robust aberration detection with as little as 10% of recommended FFPE DNA input.** FFPE sample GBM2 (Figure 6D–H) hybridized to Agilent 1 M arrays using 100% (2.0 µg), 75% (1.5 µg), 50% (1.0 µg), 25% (0.5 µg), and 10% (0.2 µg) of the recommended DNA input. Aberration analysis utilized Agilent Genomic Workbench 6.5 algorithm ADM-2 (threshold = 7.0, probes ≥7, minimum average absolute log_2_ ratio ≥0.35). A) Whole genome representation of aberrations detected (colored lines above and below x-axis) in Agilent 1 M aCGH data produced from varying DNA inputs. B) Summary of detected aberrations reveals a ∼96% (26/27) concordance between aberrations detected using 10% of standard DNA input and 100% of standard DNA input, though disparities in interval breakpoints increase significantly with lower amounts of input DNA. C) Chromosome 1 log_2_ ratios plotted for five Agilent 1 M arrays of FFPE GBM specimen GBM2 processed using the FSM ULS protocol and decreasing DNA inputs (green;log_2_ratio<−0.3, black;−0.3≤log_2_ratio≤0.3, red;log_2_ratio>0.3). While higher dLRsd indicates poorer quality in the 25% and 10% input arrays, similar aberrations (colored lines above and below x-axis) detected in the higher DNA input arrays suggest the utility of limited DNA inputs when detection of very focal (<100 kb) copy number alterations and precise breakpoints is not necessary.
(TIF)

Figure S4 **Effect of FSM ULS protocol and DNA input on Agilent 1** **M aCGH probe level sensitivity and specificity.** Data generated from FFPE sample GBM2 (Figure 6D–H) and Agilent 1 M arrays using 100% (2.0 µg), 75% (1.5 µg), 50% (1.0 µg), 25% (0.5 µg), and 10% (0.2 µg) of the recommended FFPE DNA input. Agilent Genomic Workbench 6.5 algorithm ADM-2 (threshold = 7.0, probes ≥7, minimum average absolute log_2_ ratio ≥0.35) utilized to define regions of single copy gain (0.35≤ average log_2_ ratio ≤0.58), single copy loss (−1.0≤ average log_2_ ratio ≤−0.35), and non-aberrant copy neutral regions in GBM2 FSM extended hybridization data (figure 6G) which were then used to standardize receiver operating characteristic (ROC) analysis. A,C) ROC curves plot sensitivity and 1-specificity across a range of log_2_ ratio thresholds and demonstrate that probe values in regions of either single copy gain (A) or single copy loss (C) are more readily distinguished from probe values in copy neutral regions with greater DNA input (AUC indicates area under respective ROC curve). B,D) Given ROC optimized log_2_ ratio thresholds (dashed lines) for detecting single copy gain (B) or single copy loss (D) in data from each DNA input, log_2_ ratio frequency distributions are plotted for probes in copy neutral regions and either regions of single copy gain (B) or single copy loss (D). False positive rates (FPR) and false negative rates (FNR) are calculated as follows: FPR is defined as proportion of probe values in copy neutral regions incorrectly classified as aberrant, FNR is defined as proportion of probe values in regions of gain or loss incorrectly classified as copy neutral. While the added information of genomic location and measurements from multiple probes enable algorithmic aberration detection with similar results across all DNA inputs (see Figure S3), significantly higher probe level FPR and FNR are observed at lower DNA inputs and indicate compromised array level resolution.
(TIF)

Table S1 **Summary of samples used in the study.**
(XLSX)

Table S2 **Additional QC metrics data.**
(XLSX)
