# Supplementary figures and images for: DNA Fragmentation Simulation Method (FSM) and Fragment Size Matching Improve aCGH Performance of FFPE Tissues
Source: PLoS One. 2012 Jun 15;7(6):e38881. doi: 10.1371/journal.pone.0038881 (PMC3376148; doi:10.1371/journal.pone.0038881)

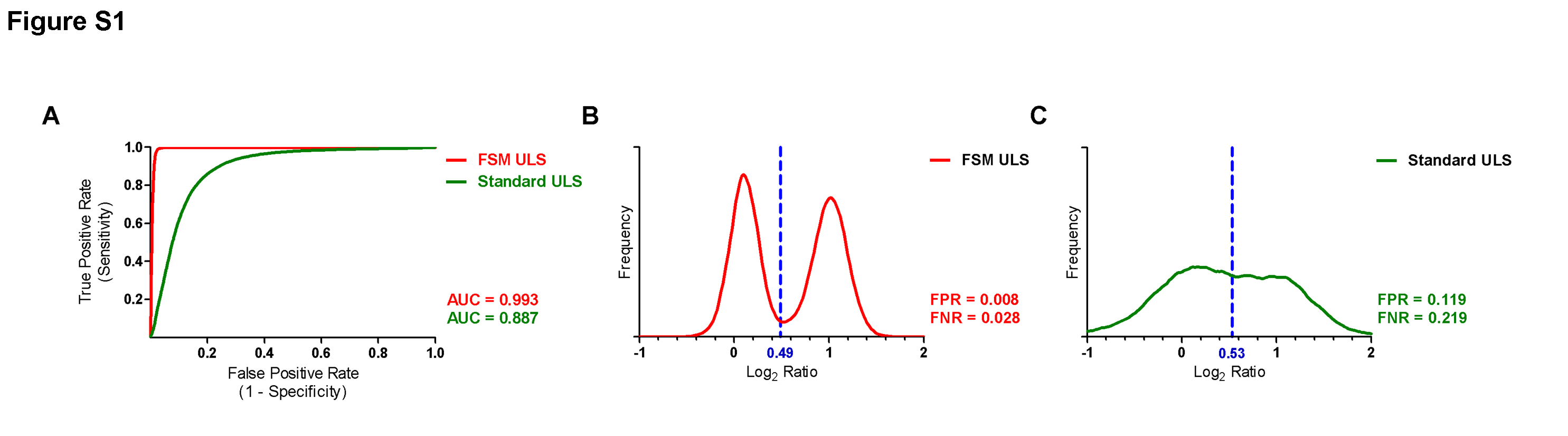

Supplement: Figure S1 — FSM ULS probe level data demonstrates greater sensitivity and specificity than Standard ULS probe level data. Female FFPE tumor DNA from sample GBM1 hybridized with normal male reference DNA (Promega) on Agilent 1 M arrays using either the FSM ULS or Standard ULS protocols. Log2 ratio data from X chromosome (XX/XY) and chromosome 8 (copy neutral) are compared for each array. A) Receiver operating characteristic (ROC) curves plot sensitivity and specificity across a range of log2 ratio thresholds and indicate aberrant (X chromosome) probe values are more readily distinguished from non-aberrant (chromosome 8) probe values in FSM ULS data than in Standard ULS data (AUC indicates area under respective ROC curve). B,C) Given optimized log2 ratio thresholds defined by ROC analysis (blue), log2 ratio frequency distributions are plotted and false positive rate (FPR) and false negative rate (FNR) are calculated. FPR is defined as proportion of copy neutral (chr8) probe values incorrectly classified as aberrant and FNR is defined as proportion of aberrant (Xchr) probe values incorrectly classified as copy neutral. (TIF) [file pone.0038881.s001.tif]

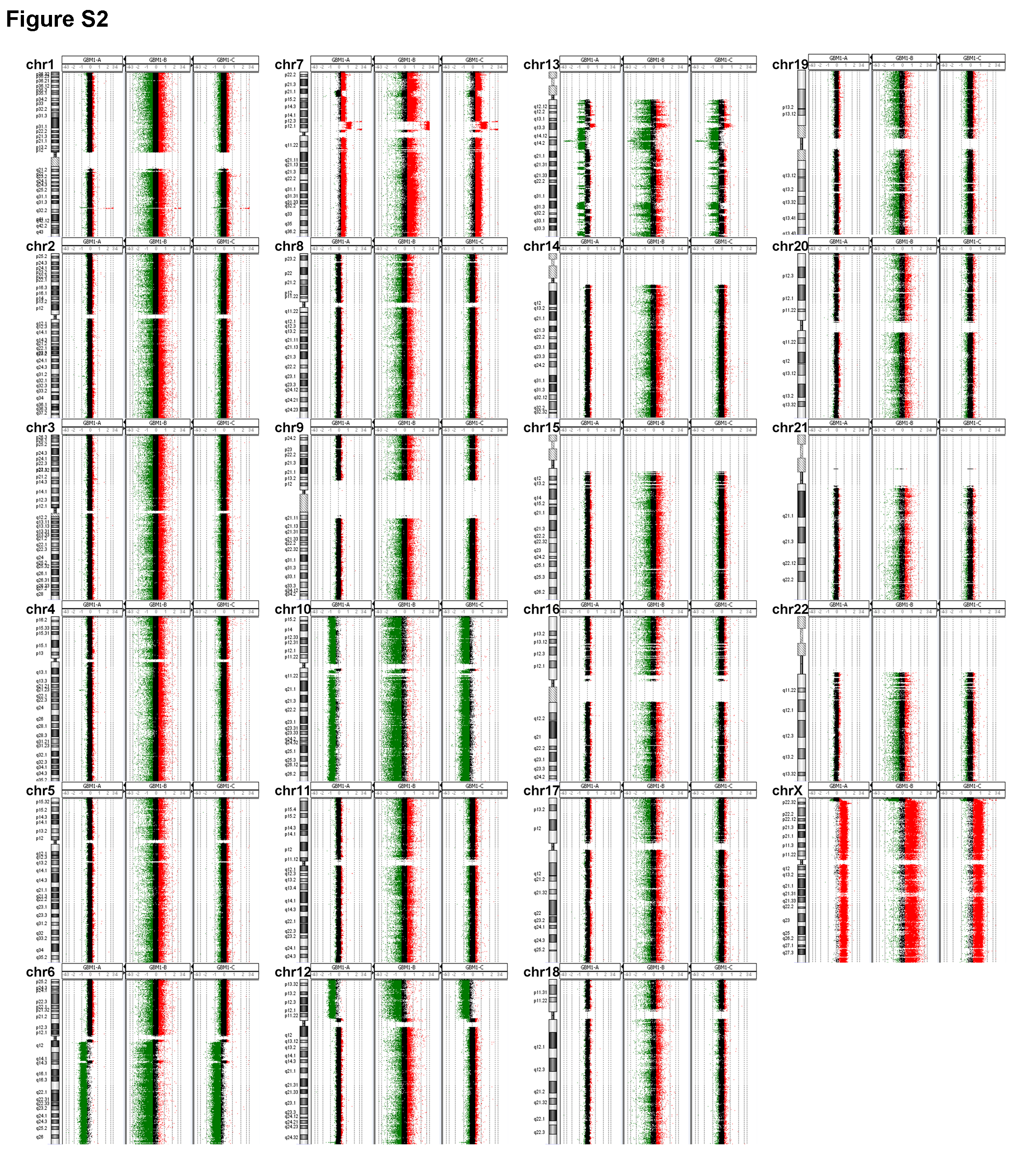

Supplement: Figure S2 — Whole genome view of Agilent 1 M array data for FFPE sample GBM1 prepared by FSM versus standard ULS methods. Log2 ratios plotted for three Agilent 1 M arrays hybridized using either the FSM ULS protocol (left), the standard ULS protocol (middle), or the FSM ULS protocol and DNA extracted with reduced duration Proteinase K digestion (right) as in Figure 6A–C (green; log2ratio<−0.3, black;−0.3≤log2ratio≤0.3, red;log2ratio>0.3). FSM methods yield lower noise across the whole genome compared to standard ULS even with shorter Proteinase K digestion. (TIF) [file pone.0038881.s002.tif]

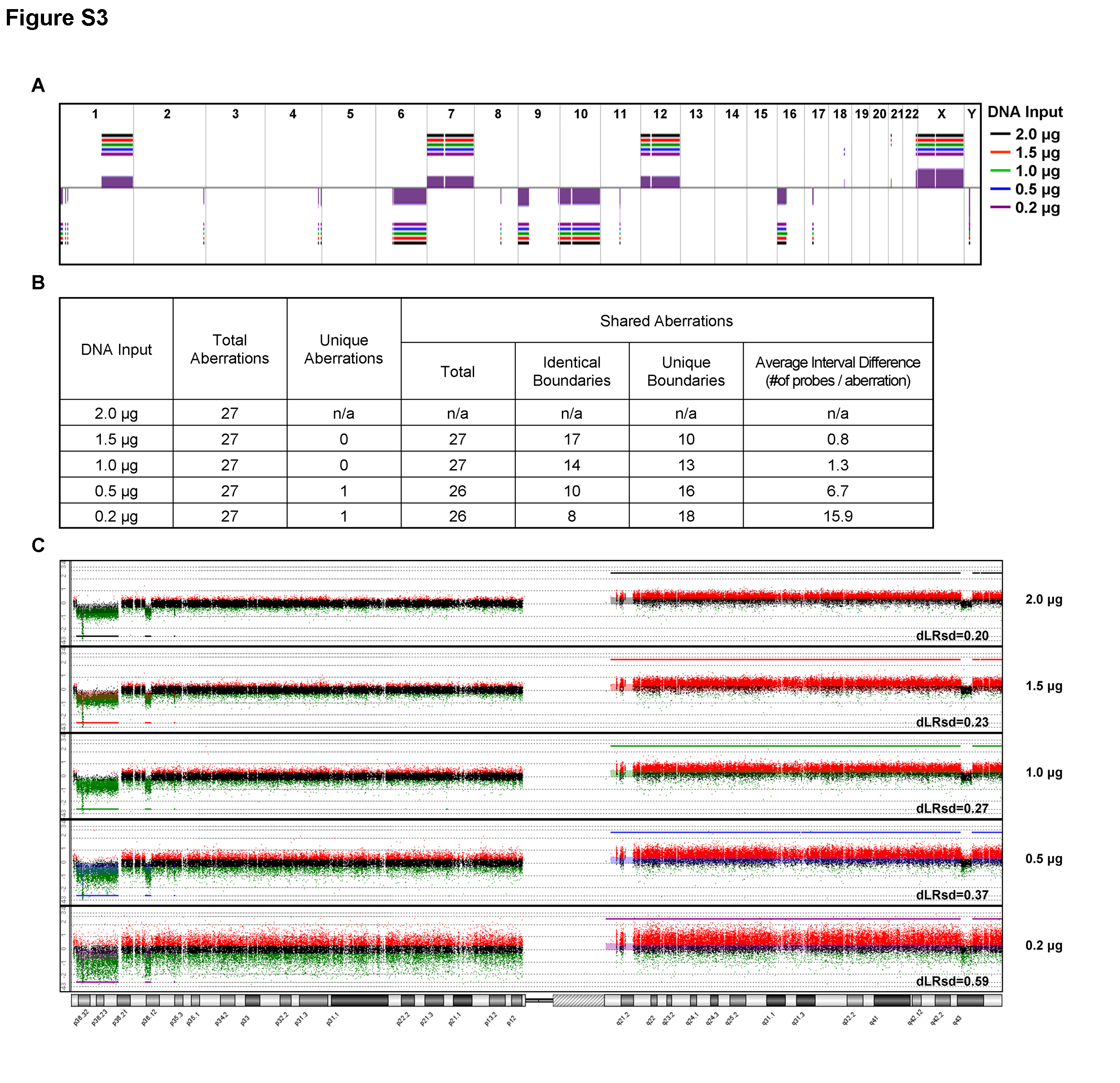

Supplement: Figure S3 — FSM ULS protocol enables robust aberration detection with as little as 10% of recommended FFPE DNA input. FFPE sample GBM2 (Figure 6D–H) hybridized to Agilent 1 M arrays using 100% (2.0 µg), 75% (1.5 µg), 50% (1.0 µg), 25% (0.5 µg), and 10% (0.2 µg) of the recommended DNA input. Aberration analysis utilized Agilent Genomic Workbench 6.5 algorithm ADM-2 (threshold = 7.0, probes ≥7, minimum average absolute log2 ratio ≥0.35). A) Whole genome representation of aberrations detected (colored lines above and below x-axis) in Agilent 1 M aCGH data produced from varying DNA inputs. B) Summary of detected aberrations reveals a ∼96% (26/27) concordance between aberrations detected using 10% of standard DNA input and 100% of standard DNA input, though disparities in interval breakpoints increase significantly with lower amounts of input DNA. C) Chromosome 1 log2 ratios plotted for five Agilent 1 M arrays of FFPE GBM specimen GBM2 processed using the FSM ULS protocol and decreasing DNA inputs (green;log2ratio<−0.3, black;−0.3≤log2ratio≤0.3, red;log2ratio>0.3). While higher dLRsd indicates poorer quality in the 25% and 10% input arrays, similar aberrations (colored lines above and below x-axis) detected in the higher DNA input arrays suggest the utility of limited DNA inputs when detection of very focal (<100 kb) copy number alterations and precise breakpoints is not necessary. (TIF) [file pone.0038881.s003.tif]

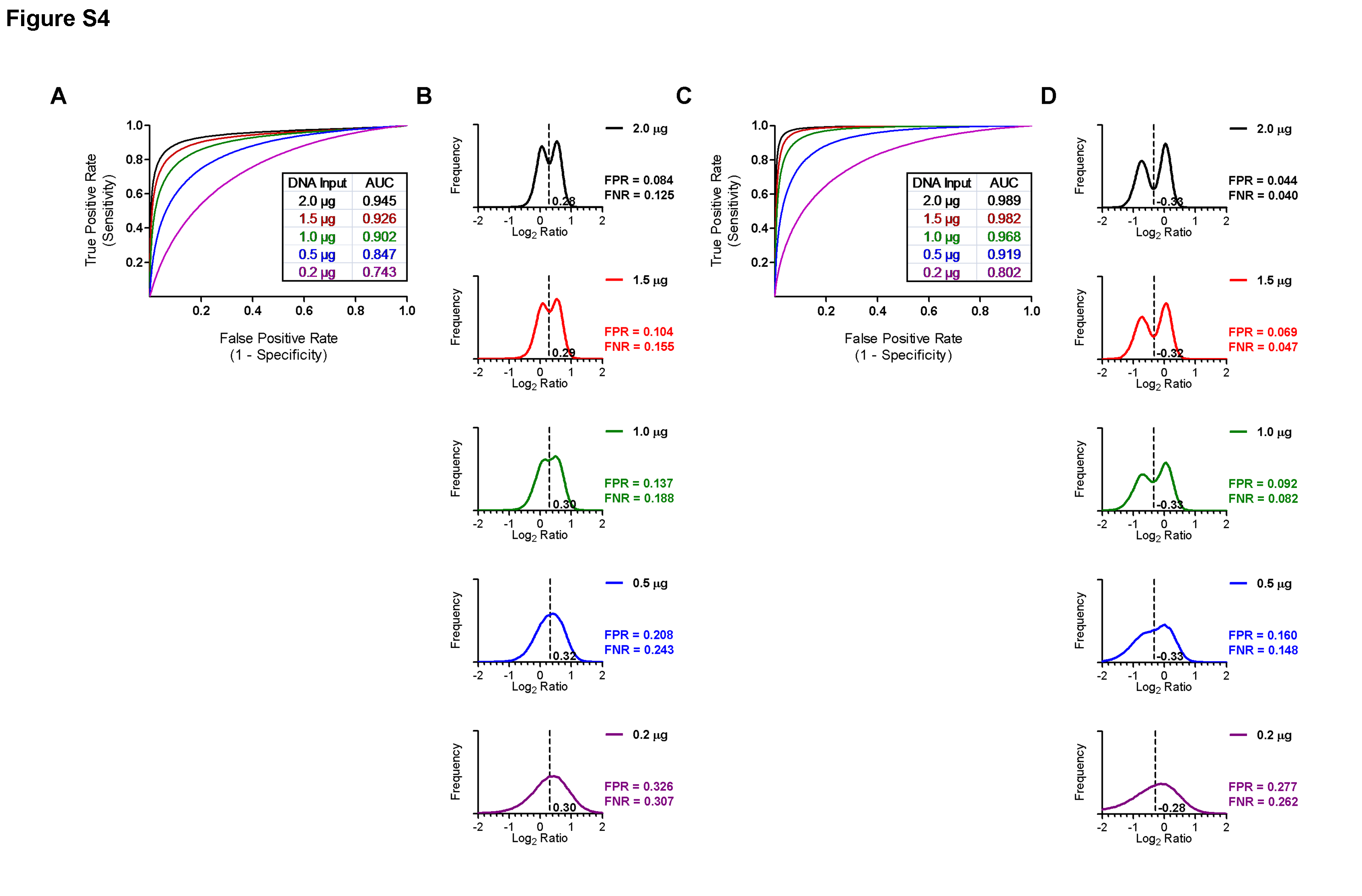

Supplement: Figure S4 — Effect of FSM ULS protocol and DNA input on Agilent 1 M aCGH probe level sensitivity and specificity. Data generated from FFPE sample GBM2 (Figure 6D–H) and Agilent 1 M arrays using 100% (2.0 µg), 75% (1.5 µg), 50% (1.0 µg), 25% (0.5 µg), and 10% (0.2 µg) of the recommended FFPE DNA input. Agilent Genomic Workbench 6.5 algorithm ADM-2 (threshold = 7.0, probes ≥7, minimum average absolute log2 ratio ≥0.35) utilized to define regions of single copy gain (0.35≤ average log2 ratio ≤0.58), single copy loss (−1.0≤ average log2 ratio ≤−0.35), and non-aberrant copy neutral regions in GBM2 FSM extended hybridization data (figure 6G) which were then used to standardize receiver operating characteristic (ROC) analysis. A,C) ROC curves plot sensitivity and 1-specificity across a range of log2 ratio thresholds and demonstrate that probe values in regions of either single copy gain (A) or single copy loss (C) are more readily distinguished from probe values in copy neutral regions with greater DNA input (AUC indicates area under respective ROC curve). B,D) Given ROC optimized log2 ratio thresholds (dashed lines) for detecting single copy gain (B) or single copy loss (D) in data from each DNA input, log2 ratio frequency distributions are plotted for probes in copy neutral regions and either regions of single copy gain (B) or single copy loss (D). False positive rates (FPR) and false negative rates (FNR) are calculated as follows: FPR is defined as proportion of probe values in copy neutral regions incorrectly classified as aberrant, FNR is defined as proportion of probe values in regions of gain or loss incorrectly classified as copy neutral. While the added information of genomic location and measurements from multiple probes enable algorithmic aberration detection with similar results across all DNA inputs (see Figure S3), significantly higher probe level FPR and FNR are observed at lower DNA inputs and indicate compromised array level resolution. (TIF) [file pone.0038881.s004.tif]
